# Lower Serum Selenoprotein P Levels Are Associated with Aortic Stiffness in Patients Receiving Maintenance Hemodialysis

**DOI:** 10.3390/diagnostics16162650

**Published:** 2026-08-20

**Authors:** Chiu-Huang Kuo, Chih-Hsien Wang, Yu-Li Lin, Yu-Hsien Lai, Chi-Chong Tang, Bang-Gee Hsu

**Affiliations:** 1 Division of Nephrology, Hualien Tzu Chi Hospital, Buddhist Tzu Chi Medical Foundation, Hualien 97004, Taiwan; 2 School of Post-Baccalaureate Chinese Medicine, Tzu Chi University, Hualien 97004, Taiwan; 3 School of Medicine, Tzu Chi University, Hualien 97004, Taiwan; 4 Department of Pharmacology, School of Medicine, Tzu Chi University, Hualien 97004, Taiwan

**Keywords:** selenoprotein P, aortic stiffness, carotid-femoral pulse wave velocity, hemodialysis, C-reactive protein

## Abstract

**Background/Objectives**: Selenoprotein P (SePP) has been linked to cardiovascular risk, but its relationship with aortic stiffness in patients on maintenance hemodialysis (MHD) is unclear. This study examined that association. **Methods**: In this cross-sectional study of 138 patients on MHD, serum SePP levels were measured with enzyme-linked immunosorbent assay, and carotid-femoral pulse wave velocity (cfPWV) was measured using the SphygmoCor system. Aortic stiffness was defined as a cfPWV of >10 m/s. Associations of SePP with cfPWV and aortic stiffness were assessed by correlation, linear regression, and penalized logistic regression. **Results**: Fifty-six patients (40.6%) had aortic stiffness and had lower serum SePP levels than those without aortic stiffness (*p* = 0.001). Patients with aortic stiffness were older (*p* = 0.037) and had higher systolic blood pressure (*p* = 0.005), higher glucose (*p* = 0.021) and C-reactive protein (*p* = 0.021) levels, and a higher prevalence of diabetes mellitus (*p* = 0.034) and hypertension (*p* = 0.006). Log-transformed SePP was independently and inversely associated with log-transformed cfPWV in both the forward stepwise and forced-entry models (*p* < 0.001 for both). By Spearman’s analysis, SePP was negatively correlated with cfPWV (*p* < 0.001). After adjustment for significant covariates, higher SePP levels remained independently associated with lower odds of aortic stiffness in multivariable and penalized logistic regression models. **Conclusions**: Lower serum SePP levels were independently associated with higher cfPWV and greater odds of aortic stiffness in patients receiving MHD.

## 1. Introduction

Selenoprotein P (SePP), synthesized primarily in the liver, is the principal selenium-containing protein in plasma that facilitates selenium transport to various tissues, particularly the brain and testes [1,2,3]. Under physiological conditions, SePP supports antioxidant enzyme activity, maintains redox homeostasis, and contributes to vascular protection [4], whereas elevated SePP levels can inhibit AMP-activated protein kinase and disrupt insulin signaling, potentially resulting in insulin resistance and metabolic dysfunction in pathologic states, such as diabetes mellitus [5,6]. During severe illness, SePP levels decline due to the downregulation of hepatic SePP synthesis by inflammatory cytokines [7,8]. SePP acts as both an antioxidant and a metabolic regulator: Adequate SePP levels are beneficial, whereas excess or deficiency can cause harm.

Extensive evidence indicates that selenium status and the levels of specific selenoproteins, most notably SePP, the principal selenium transport protein, and glutathione peroxidase, a key antioxidant selenoenzyme, are closely associated with cardiovascular disease (CVD) risk. Selenium contributes to key cardiovascular processes via the synthesis of selenoproteins, which play critical roles in cardiovascular integrity, including the regulation of oxidative stress, redox balance, thyroid hormone metabolism, and calcium homeostasis [9]. Selenium deficiency impairs the synthesis of specific selenoproteins, leading to increased oxidative stress and inflammation and compromised vascular function, thereby increasing CVD risk. Adequate selenium intake may attenuate the progression of atherosclerosis by supporting these protective mechanisms and mitigating damage mediated by low-density lipoprotein oxidation and inflammation [10]. As the primary selenium transporter, SePP enhances antioxidant enzyme activity and can directly neutralize reactive oxygen and nitrogen species in tissues, thereby reducing oxidative injury [11].

Large-scale epidemiologic studies examining the association of SePP with cardiovascular health and mortality reveal a complex relationship. Numerous observational studies have demonstrated that higher selenium levels, assessed by blood or toenail measurements, are associated with a reduced risk of coronary heart disease [12]. For example, a Swedish cohort study involving 4366 participants reported lower all-cause and cardiovascular mortality in individuals with elevated SePP levels, with those in the highest quartile experiencing approximately half the cardiovascular mortality rate of those in the lowest quartile [11]. Similarly, in a large-scale, statewide, population-based prospective cohort study of older adults aged 50–74 years conducted in Germany, lower SePP levels were associated with a significantly higher mortality rate, and the risk of cardiovascular death was 24% higher in participants with the lowest SePP levels than in those with the highest SePP levels [13]. Additionally, a cross-sectional analysis of the US National Health and Nutrition Examination Survey data found that a higher dietary selenium intake was associated with a lower prevalence of hypertension, suggesting that adequate selenium intake may mitigate certain cardiovascular risk factors [14]. Beyond biochemical endpoints, selenium status has also been linked to arterial stiffness itself in non-dialysis populations: lower selenium intake has been associated with higher brachial-ankle pulse wave velocity in individuals at high cardiovascular risk, and lower baseline serum selenium has predicted greater cfPWV progression over 10 years in a general-population cohort [15,16], although comparable data specifically relating serum SePP to aortic stiffness are lacking, particularly in patients on maintenance hemodialysis (MHD).

CVD is the leading cause of death among patients with end-stage kidney disease, with both incidence and mortality rates far exceeding those observed in the general population. Studies have shown that cardiovascular mortality is 10- to 30-fold higher in patients on dialysis than in age and sex-matched individuals in the general population and that this disparity may reach 500-fold in younger patients [17]. Beyond traditional risk factors, arterial stiffening is a central pathologic process in the development of CVD. Carotid-femoral pulse wave velocity (cfPWV), the gold standard for measuring large artery stiffness, is an independent predictor of cardiovascular events and all-cause mortality in patients on dialysis, and its predictive value remains robust even after adjustment for age, sex, high blood pressure, diabetes mellitus, and other factors [18].

Selenium deficiency frequently occurs in patients with chronic kidney disease (CKD), particularly in those with advanced-stage disease and those undergoing hemodialysis [19]. In patients with end-stage kidney disease, dietary limitations and malnutrition, combined with the loss of trace elements during dialysis, increase susceptibility to selenium deficiency [19]. In patients on regular hemodialysis, chronic uremic toxin accumulation, persistent low-grade inflammation, oxidative stress imbalance, and disrupted calcium-phosphorus metabolism contribute to structural and functional alterations in arterial walls. Given that SePP reduces oxidative stress and inflammation as the principal selenium transport protein and antioxidant, insufficient SePP levels may promote arterial stiffening. Studies in CKD cohorts have demonstrated a positive correlation between serum selenium levels and kidney function markers, such as estimated glomerular filtration rate, whereas data from the US National Health and Nutrition Examination Survey indicate that patients with CKD in the highest quartile of serum selenium levels have approximately half the cardiovascular mortality risk compared with those in the lowest quartile [20]. Selenium supplementation trials in patients on dialysis have primarily targeted biochemical endpoints, including increased serum selenium levels and glutathione peroxidase activity, reduced DNA oxidative damage, and decreased C-reactive protein (CRP) levels, whereas the impact of selenium supplementation on clinical outcomes, such as cardiovascular events and arterial structural changes, remains inconclusive [19]. A recent meta-analysis of seven randomized controlled trials involving 2080 patients on MHD found that selenium supplementation significantly increased serum selenium levels but did not substantially improve CRP and hemoglobin levels or the lipid profile [21]. Notably, no trial to date has evaluated arterial stiffness as an endpoint for selenium supplementation. Moreover, no study to date has elucidated the relationship between serum SePP levels and aortic stiffness in patients on MHD, which represents a critical gap in current knowledge. The present study aimed to examine the relationship of serum SePP levels with aortic stiffness, evaluated by cfPWV, in patients on MHD.

## 2. Materials and Methods

### 2.1. Study Design and Participant Enrollment

Patients undergoing MHD at Hualien Tzu Chi Hospital between March and October 2023 who met the eligibility criteria below were enrolled in this cross-sectional study; all participants were dialyzed using high-flux polysulfone FX-series membranes (Fresenius Medical Care, Bad Homburg, Germany). The sample size was determined by the number of consecutively eligible patients available at our center during the study period rather than by an a priori power calculation; a post hoc power analysis is reported in Section 3.6. Eligible participants were aged ≥20 years and were receiving dialysis for at least 6 months, undergoing regular 4-h hemodialysis sessions three times weekly. A total of 249 patients undergoing MHD were initially assessed for eligibility. Of these, 79 were excluded because of recent acute infection or inflammatory flare (*n* = 5), active malignancy (*n* = 14), limb amputation (*n* = 14), acute heart failure (*n* = 2), cirrhosis (*n* = 8), thyroid disorders requiring pharmacotherapy (*n* = 4), stroke (*n* = 8), peripheral arterial occlusive disease (*n* = 11), bedridden status (*n* = 12), or high-dose selenium supplement use within the preceding 3 months (*n* = 1). An additional 32 patients were not enrolled because they were unable to understand the study information or provide informed consent. Consequently, 138 patients were enrolled and included in the final analysis. This 3-month threshold was chosen because it exceeds the estimated turnover time of plasma SePP, allowing serum SePP concentrations to return to an individual’s supplementation-independent steady state and thereby avoiding confounding by exogenous selenium intake. Demographic and clinical data, including diabetes mellitus and hypertension status, were obtained from the medical records. Dialysis adequacy was assessed using the single-pool fractional urea clearance index (Kt/V) and the urea reduction ratio, calculated from pre- and post-hemodialysis urea levels based on a standard single-compartment kinetic model. All study assessments, including blood sampling, anthropometric measurement, and cfPWV measurement, were performed once for each participant, on the same midweek pre-dialysis day, during the study enrollment period.

### 2.2. Anthropometric and Laboratory Assessments

For each participant, height was recorded and body weight measured immediately before and after the hemodialysis session, from which body mass index (weight in kg divided by height in m squared) was derived. On a midweek dialysis day, pre-dialysis venous blood was drawn into both ethylenediaminetetraacetic acid and serum-separator tubes. Hemoglobin was quantified from the ethylenediaminetetraacetic acid-anticoagulated sample on a Sysmex XS-1000i analyzer (Sysmex America, Mundelein, IL, USA), while the serum-tube sample was centrifuged at 3000× *g* and the resulting serum used for biochemical profiling, including albumin, total cholesterol, triglycerides, blood urea nitrogen, creatinine, glucose, calcium, phosphorus, and CRP, on a Siemens Advia 1800 autoanalyzer (Siemens Healthcare, Erlangen, Germany). Intact parathyroid hormone (iPTH) and SePP were quantified by enzyme-linked immunosorbent assay (iPTH kit cat. no. NM59041, IBL International, Hamburg, Germany; SePP kit cat. no. CSB-EL021018HU, Cosmo Bio USA, Carlsbad, CA, USA), with intra-/interassay coefficients of variation of 4.2%/3.6% for iPTH and 4.6%/6.2% for SePP.

### 2.3. Blood Pressure and Carotid-Femoral Pulse Wave Velocity Measurements

After participants rested supine for at least 10 min in a quiet, temperature-controlled room, brachial systolic and diastolic pressures were recorded three times at 5-min intervals with an automated oscillometric device; the mean of the three readings was used in subsequent analyses.

A trained operator acquired cfPWV with the SphygmoCor XCEL system (AtCor Medical, Sydney, NSW, Australia; software version 1.30), a validated device that captures carotid and femoral pulse waveforms simultaneously and noninvasively, the carotid signal via a high-fidelity tonometer positioned over the carotid artery, and the femoral signal via a pneumatic thigh cuff. Participants remained still and refrained from speaking throughout each measurement to limit motion-related artifact [22,23].

Path length was obtained by the subtraction method, namely the sternal-notch-to-femoral-cuff distance minus the sternal-notch-to-carotid distance, with all distances measured to the nearest millimeter using a flexible tape. cfPWV was then calculated automatically as this path length divided by the carotid-femoral pulse transit time, averaged across several consecutive cardiac cycles; recordings with poor signal quality or excessive pulse-to-pulse variability were repeated until a stable trace was obtained.

To avoid the acute hemodynamic effects of dialysis, cfPWV was recorded pre-dialysis on a midweek session day. Following the 2018 European Society of Cardiology/European Society of Hypertension guidelines’ threshold of >10 m/s for clinically significant aortic stiffness, participants were accordingly grouped into aortic-stiffness and control categories [24].

### 2.4. Statistical Analyses

No participant had missing data for any of the clinical, biochemical, or hemodynamic variables analyzed in this study. Variable normality was first checked with the Kolmogorov–Smirnov test. Continuous data are reported as mean ± standard deviation when normally distributed and as median (interquartile range) otherwise; categorical data are reported as counts and percentages. Group differences in continuous variables were tested with Student’s *t*-test (normal distribution) or the Mann–Whitney *U* test (non-normal distribution), and differences in categorical variables were tested with the chi-square test.

Variables with skewed distributions, including MHD duration, cfPWV, albumin, triglycerides, glucose, iPTH, CRP, and SePP, were log_10_-transformed before subsequent parametric analyses. The association of log-transformed cfPWV with clinical parameters was first examined using Pearson’s correlation analysis. Next, variables significantly associated with log-transformed cfPWV were evaluated using forward stepwise and forced-entry multiple linear regression models to identify independent determinants of cfPWV.

Aortic stiffness was analyzed as a binary dependent variable, defined as a cfPWV of >10 m/s. Multivariable logistic regression analysis was performed to evaluate the associations between clinical variables and aortic stiffness. Covariates included factors significantly associated with aortic stiffness status, namely, diabetes mellitus, hypertension, age, SBP, glucose, CRP, and SePP. Additionally, to assess the robustness of the findings and reduce the potential risk of model overfitting, penalized logistic regression analyses, including LASSO, Ridge, and Elastic Net regression, were performed using the same covariates. Bootstrap resampling with 400 iterations was applied for internal validation. The optimal penalty parameter was selected using 10-fold cross-validation, and predictors were standardized before model fitting.

The discrimination and calibration performance of SePP in identifying aortic stiffness was evaluated using ROC curve analysis and logistic calibration assessment. The AUC was calculated to assess the discriminatory ability of SePP to detect aortic stiffness. The optimal SePP cutoff value was determined using the Youden index, and the corresponding sensitivity and specificity were calculated. Calibration performance was assessed using the Brier score and the Hosmer-Lemeshow goodness-of-fit test. Internal validation was performed using stratified bootstrap resampling with 400 iterations to obtain bootstrap-corrected estimates of the AUC, Brier score, calibration intercept, and calibration slope.

Spearman’s rank correlation coefficients were used to examine the relationship of serum SePP levels with clinical parameters. All statistical analyses were performed using SPSS for Windows (version 25.0; IBM, Armonk, NY, USA), and R statistical software (version 4.2.2; R Foundation for Statistical Computing, Vienna, Austria). A two-sided *p*-value of <0.05 was considered statistically significant.

### 2.5. Ethical Considerations

This study was conducted in accordance with the Declaration of Helsinki and was approved by the Institutional Review Board of Hualien Tzu Chi Hospital (approval no. IRB108-219-A; approval granted on 19 November 2019). Written informed consent was obtained from all participants prior to enrollment.

## 3. Results

### 3.1. Clinical Characteristics According to Aortic Stiffness Status

In the study cohort of 138 patients on MHD, 56 (40.6%) had aortic stiffness (cfPWV of >10 m/s). Compared with the patients without aortic stiffness (i.e., the control group), those with aortic stiffness were older (*p* = 0.037) with higher systolic blood pressure (SBP, *p* = 0.005), glucose (*p* = 0.021), and CRP (*p* = 0.021) levels. The prevalence of diabetes mellitus and hypertension was also significantly higher in the aortic stiffness group than in the control group (*p* = 0.034 and *p* = 0.006, respectively), whereas serum SePP levels were significantly lower in the aortic stiffness group than in the control group (*p* = 0.001). Hemodialysis duration, body mass index, lipid profile, dialysis-related parameters, dialysis adequacy indices, and medication use were not significantly different between the two groups (Table 1).

**Table 1:** Baseline clinical characteristics of patients stratified by aortic stiffness status.

Parameters	All Patients (*n* = 138)	Control Group (*n* = 82)	Aortic Stiffness Group (*n* = 56)	*p* Value
Age (years)	62.87 ± 13.11	61.18 ± 13.70	65.88 ± 11.42	0.037 *
HD duration (months)	56.52 (23.40–123.60)	71.88 (21.69–136.80)	49.08 (26.67–87.24)	0.252
Height (cm)	160.14 ± 8.38	159.29 ± 8.51	161.39 ± 8.10	0.149
Pre-HD body weight (kg)	64.72 ± 14.86	63.75 ± 15.76	66.16 ± 13.42	0.355
Post-HD body weight (kg)	61.77 ± 14.18	60.99 ± 14.94	62.92 ± 13.04	0.436
Body mass index (kg/m^2^)	25.05 ± 4.86	24.98 ± 5.29	25.15 ± 4.18	0.838
Carotid-femoral PWV (m/s)	9.05 (7.60–12.33)	7.80 (7.18–8.80)	12.90 (11.83–15.38)	<0.001 *
Systolic blood pressure (mmHg)	138.49 ± 21.01	134.38 ± 21.90	144.52 ± 18.17	0.005 *
Diastolic blood pressure (mmHg)	76.25 ± 15.07	76.16 ± 14.91	76.38 ± 15.44	0.934
Hemoglobin (g/dL)	10.41 ± 1.10	10.30 ± 1.21	10.58 ± 0.88	0.139
Albumin (g/dL)	4.10 (3.90–4.40)	4.10 (3.90–4.40)	4.10 (4.00–4.30)	0.564
Total cholesterol (mg/dL)	143.71 ± 34.64	144.68 ± 38.58	139.36 ± 27.65	0.224
Triglyceride (mg/dL)	117.50 (85.50–182.00)	112.00 (83.75–200.75)	124.50 (89.25–175.00)	0.828
Glucose (mg/dL)	131.00 (108.50–155.00)	128.50 (103.75–143.25)	136.00 (115.25–176.50)	0.021 *
Blood urea nitrogen (mg/dL)	61.38 ± 14.94	60.90 ± 14.44	62.07 ± 15.74	0.653
Creatinine (mg/dL)	9.41 ± 2.07	9.49 ± 2.05	9.29 ± 2.12	0.584
Total calcium (mg/dL)	9.07 ± 0.73	9.00 ± 0.71	9.16 ± 0.74	0.207
Phosphorus (mg/dL)	4.69 ± 1.27	4.69 ± 1.29	4.69 ± 1.25	0.990
C-reactive protein (mg/dL)	0.29 (0.08–0.66)	0.23 (0.07–0.53)	0.41 (0.11–0.83)	0.021 *
iPTH (pg/mL)	193.15 (58.50–360.33)	225.50 (95.50–440.68)	149.35 (47.83–340.50)	0.145
SePP (mg/L)	3.59 (2.53–5.08)	4.08 (2.75–5.86)	2.94 (2.35–4.27)	0.001 *
Urea reduction rate	0.73 ± 0.04	0.74 ± 0.05	0.73 ± 0.04	0.320
Kt/V (Gotch)	1.34 ± 0.17	1.35 ± 0.18	1.32 ± 0.16	0.266
Female, *n* (%)	67 (48.6)	42 (51.2)	25 (44.6)	0.448
Diabetes mellitus, *n* (%)	59 (42.8)	29 (35.4)	30 (53.6)	0.034 *
Hypertension, *n* (%)	82 (59.4)	41 (50.0)	41 (73.2)	0.006 *
ARB, *n* (%)	34 (24.6)	17 (20.7)	17 (30.4)	0.198
β-blocker, *n* (%)	39 (28.3)	20 (24.4)	19 (33.9)	0.222
Calcium channel blocker, *n* (%)	38 (27.5)	22 (26.8)	16 (28.6)	0.822
Statin, *n* (%)	28 (20.3)	15 (18.3)	13 (23.2)	0.480
Fibrate, *n* (%)	30 (21.7)	20 (24.4)	10 (17.9)	0.361

Values for continuous variables are shown as mean ± standard deviation after analysis by Student’s *t*-test; variables not normally distributed are shown as median and interquartile range after analysis by the Mann–Whitney U test; values are presented as number (%) and analyzed by the chi-square test. HD, hemodialysis; PWV, pulse wave velocity; SePP, selenoprotein P; Kt/V, fractional clearance index for urea; iPTH, intact parathyroid hormone; ARB, angiotensin receptor blocker. * *p* < 0.05 was considered statistically significant.

### 3.2. Factors Associated with Aortic Stiffness

By Pearson correlation analysis, age (*r* = 0.203, *p* = 0.017), SBP (*r* = 0.180, *p* = 0.034), log-transformed glucose (*r* = 0.189, *p* = 0.027), log-transformed CRP (*r* = 0.246, *p* = 0.004), and log-transformed SePP (*r* = −0.437, *p* < 0.001) were significantly associated with log-transformed cfPWV. These variables were included in multivariable forward stepwise and forced-entry linear regression models for adjustment. In the multivariable stepwise linear regression model, age (β = 0.163, *p* = 0.032), log-transformed CRP (β = 0.183, *p* = 0.017), and log-transformed SePP (β = −0.420, *p* < 0.001) remained independently associated with log-transformed cfPWV. In the multivariable forced-entry linear regression model, log-transformed SePP remained independently and inversely associated with log-transformed cfPWV (β = −0.409, *p* < 0.001), together with age (β = 0.179, *p* = 0.022) and log-transformed CRP (β = 0.162, *p* = 0.034). The multivariable stepwise and forced-entry linear regression models explained 26.2% and 28.6% of the variance in log-transformed cfPWV, respectively (Table 2).

### 3.3. Multivariable and Penalized Logistic Regression Analyses for Aortic Stiffness

Multivariable logistic regression analysis, adjusted for significant factors (*p* < 0.05), including diabetes mellitus, hypertension, age, SBP, glucose, CRP, and SePP (Table 3), revealed that higher SePP levels were independently associated with lower odds of aortic stiffness. Specifically, each 1 mg/L increase in SePP was associated with a 32.4% lower likelihood of aortic stiffness (odds ratio [OR] 0.676, 95% confidence interval [CI] 0.535–0.853; *p* < 0.001). Additionally, age (OR 1.038, 95% CI 1.002–1.074; *p* = 0.036), SBP (OR 1.036, 95% CI 1.000–1.072; *p* = 0.050), and CRP (per 0.1 mg/dL increase) (OR 1.077, 95% CI 1.001–1.159; *p* = 0.047) were significantly associated with aortic stiffness (Table 3).

The inverse association between SePP and aortic stiffness remained consistent across penalized logistic regression models, including the Least Absolute Shrinkage and Selection Operator (LASSO), Ridge, and Elastic Net regression models (*p* = 0.005 for all models). Additionally, SBP remained significantly associated with aortic stiffness in all three penalized models, whereas CRP remained significantly associated with aortic stiffness in the Ridge and Elastic Net regression models. Diabetes mellitus, hypertension, and glucose were not significantly associated with aortic stiffness after adjustment in these models (Table 3).

### 3.4. Discrimination and Calibration Performance of SePP for Identifying Aortic Stiffness

By receiver operating characteristic (ROC) curve analysis, SePP had moderate discriminatory ability for identifying aortic stiffness, with an area under the ROC curve (AUC) of 0.674 (95% CI 0.580–0.759; *p* < 0.001). The optimal SePP cutoff value determined by the Youden index was 3.26 mg/L, with a sensitivity of 0.571 and a specificity of 0.720. Given our analysis showing that lower SePP levels were associated with a higher likelihood of aortic stiffness, SePP levels below this cutoff indicated increased risk. The Brier score was 0.217, whereas the Hosmer-Lemeshow goodness-of-fit test revealed no significant evidence of poor calibration (*p* = 0.339) (Table 4). After internal validation using stratified bootstrap resampling with 400 iterations, the bootstrap-corrected AUC and Brier score were 0.678 and 0.219, respectively, with a bootstrap-corrected calibration intercept of 0.037 and a calibration slope of 1.105.

### 3.5. Association of SePP with Clinical Variables

By Spearman’s correlation analysis, serum SePP levels were significantly and negatively correlated with cfPWV (ρ = −0.423, *p* < 0.001), hemoglobin (ρ = −0.241, *p* = 0.004) and CRP (ρ = −0.181, *p* = 0.033) (Table 5).

### 3.6. Post Hoc Power Analysis

A post hoc power analysis was performed for the multivariable linear regression, using log-transformed cfPWV as the continuous outcome. With 138 patients, 5 predictors in the forced-entry multivariable linear regression model, a two-sided alpha level of 0.05, and 80% power, the study could detect an overall effect size of approximately f^2^ = 0.097. The observed overall effect size of the forced-entry model was f^2^ = 0.401, calculated from the model R^2^ of 0.286. As the observed effect size exceeded the minimum detectable effect size, the study had sufficient statistical power to detect the overall multivariable association between the included predictors and cfPWV.

## 4. Discussion

This study aimed to examine the relationship between serum SePP levels and aortic stiffness in patients on MHD. Consistent with this aim, in the present cross-sectional study, SePP levels were significantly lower in patients on MHD with aortic stiffness than in those without aortic stiffness, and in older patients with higher SBP, higher glucose and CRP levels, and higher rates of diabetes mellitus and hypertension. Importantly, log-transformed SePP remained independently and inversely associated with log-transformed cfPWV in both stepwise and forced-entry multivariable linear regression models. Furthermore, multivariable and penalized logistic regression analyses confirmed the independent association of higher SePP levels with lower odds of aortic stiffness. Altogether, these findings indicate that SePP deficiency may contribute to arterial stiffening in patients on MHD.

Our findings extend prior observations but contradict studies in metabolically replete populations. For example, in a Hong Kong study of individuals at high cardiovascular risk, Chan et al. found that brachial-ankle pulse wave velocity (baPWV) was significantly higher in individuals with low selenium intake than in those with adequate selenium intake, linking selenium insufficiency to increased arterial stiffness [15]. Similarly, a 10-year prospective cohort study in South Africa observed an inverse association between baseline serum selenium level and cfPWV, supporting the vasoprotective effects of selenium [16]. Additionally, a study of patients admitted for verification of obstructive sleep apnea reported that SePP levels below the study cohort-specific median (<0.64 ng/mL), rather than the general population median, were associated with less favorable blood pressure and echocardiographic parameters [25]. Together, these studies support a protective role for SePP in populations at high cardiovascular risk, consistent with our observation of inverse correlation between SePP and cfPWV. However, not all studies agree with these findings. SePP was significantly higher in people with non-alcoholic fatty liver disease (NAFLD) and visceral obesity than in those without NAFLD; SePP was also positively correlated with baPWV [6]. In patients with NAFLD, SePP was inversely correlated with flow-mediated dilation, suggesting that elevated SePP levels may negatively impact vascular function [26]. Several factors may explain these differences. First, the nutritional and metabolic backgrounds differ substantially across these studies. In individuals with obesity, NAFLD, or diabetes mellitus, SePP often rises due to metabolic dysfunction and excess secretion from the liver, aligning with poor cardiovascular outcomes. Conversely, in individuals with malnutrition, CKD, or low selenium intake, low SePP levels likely reflect weak antioxidant defenses that promote arterial stiffness. Second, methods used to assess vascular function varied across the studies. baPWV reflects peripheral arterial elasticity, which is more likely to be impacted by blood pressure and sympathetic activity, whereas cfPWV and flow-mediated dilation assess aortic stiffness and endothelial function, likely more accurately reflecting the role of SePP in cardiovascular function.

SePP is the most abundant selenium-containing protein in plasma, accounting for more than 50% of total plasma selenium content [27,28]. SePP is a functional biomarker of selenium supply that reflects tissue selenium utilization rather than the total plasma selenium level [27]. Results of studies across European cohorts in Germany and Sweden and across clinical settings, including primary prevention, older adults, and patients with heart failure [11,13,29], support SePP deficiency as an established risk factor for adverse cardiovascular outcomes and mortality. Recent studies have expanded the clinical relevance of SePP. In hospitalized patients with heart failure, lower SePP levels were independently associated with anemia and iron deficiency, suggesting a potential role for SePP in erythropoiesis and iron metabolism [30]. In the present study, SePP levels were also significantly, albeit negatively, correlated with hemoglobin and CRP levels. These findings indicate that the relationship between SePP, anemia, and inflammation is complex in patients on MHD and may differ from that observed in heart failure cohorts. Further studies are warranted to clarify the direct impact of SePP on erythropoiesis, iron metabolism, and inflammatory pathways in patients on MHD. Given that both SePP and CRP were independently associated with aortic stiffness in this cohort, a combined marker incorporating both an antioxidant/nutritional index (SePP) and an inflammatory index (CRP) may offer improved discrimination for aortic stiffness risk in patients on MHD compared with either marker alone; this composite approach warrants prospective evaluation.

Given that SePP plays antioxidant and metabolic regulatory roles in vivo and that its excess or deficiency can lead to adverse effects, multiple studies suggest that the relationship between SePP or total selenium levels and cardiovascular risk is not linear but may follow a J- or U-shaped curve, indicating an optimal range [28,31]. Some studies have found increased cardiovascular risk at both low and high selenium/SePP levels, although evidence for harm at excessive levels remains limited and variable [11,13]. Variations in the curve shape (L-, J-, or U-shaped) across studies can be attributed to differences in biomarkers, populations, and endpoints, complicating broader interpretations. Altogether, the current evidence most consistently supports risks associated with low SePP levels, whereas the potential harm of elevated SePP levels requires further investigation [13,31].

In the present study, age and CRP were also independent predictors of cfPWV, in agreement with known mechanisms of arterial stiffening. Vascular aging is characterized by elastin fragmentation, cross-linking of collagen fibers, and accumulation of advanced glycation end-products, which collectively reduce arterial compliance [32,33,34]. Chronic inflammation promotes endothelial dysfunction by downregulating nitric oxide bioavailability and upregulating vasoconstrictive mediators, while facilitating vascular smooth muscle cell proliferation and extracellular matrix remodeling [35].

SePP may contribute to arterial stiffness through several interrelated mechanisms. First, as the major plasma selenium transport protein, SePP delivers selenium to vascular tissues and supports the synthesis and activity of antioxidant selenoenzymes, including glutathione peroxidase, thereby reducing oxidative injury within the vascular wall [2,36]. Second, SePP exerts direct antioxidant effects by scavenging reactive oxygen and nitrogen species, including phospholipid hydroperoxides, which protect the vascular endothelium from oxidative damage [2,37]. Third, by maintaining redox homeostasis, SePP may preserve endothelial nitric oxide bioavailability and support normal vasodilatory function [38,39]. In patients undergoing MHD, circulating SePP levels may be reduced due to dietary restrictions, dialysis-related loss, and the uremic milieu [40,41]. Reduced SePP availability may be associated with weaker antioxidant and endothelial protective capacity, which could plausibly contribute to arterial stiffening; however, this cross-sectional study cannot establish the direction of this relationship. Additionally, a recent review of cardiorenal anemia syndrome suggested that selenoprotein dysfunction augments oxidative stress and affects erythropoiesis, calcium homeostasis, and thyroid hormone metabolism, all of which may further promote vascular dysfunction and arterial stiffening in patients with CKD [42].

The present study has several limitations. First, the cross-sectional design precludes establishing a causal relationship between SePP levels and aortic stiffness; reduced SePP levels may be a cause, a consequence, or an epiphenomenon of aortic stiffening driven by a shared underlying factor. Reverse causation is also possible, as advanced arterial stiffening and its associated systemic inflammation could themselves suppress hepatic SePP synthesis. Second, as a single-center study enrolling consecutively eligible patients, selection bias cannot be fully excluded and the generalizability of the findings to other dialysis populations warrants caution; patients who were bedridden, had active malignancy, or had acute illness were excluded, which may have selected for a relatively clinically stable cohort and could bias the observed SePP-aortic stiffness association toward the null (i.e., underestimate its true magnitude in an unselected dialysis population). Third, the relatively small sample size limited statistical power for subgroup analyses, although a post hoc power analysis (Section 3.6) indicated adequate power for the primary multivariable model; the small cohort size also precluded further sensitivity analyses, such as excluding specific patient subgroups or applying alternative covariate selection strategies. Fourth, although we adjusted for the major clinical confounders significantly associated with aortic stiffness in this cohort—diabetes mellitus, hypertension, age, SBP, glucose, and CRP—and confirmed robustness using LASSO, Ridge, and Elastic Net penalized regression, the possibility of unmeasured residual confounding (e.g., dietary selenium intake, medication adherence, or subclinical inflammation not captured by CRP) cannot be excluded. Fifth, we did not measure the levels of other selenoproteins, such as glutathione peroxidase, or total selenium levels, which precluded a comprehensive assessment of selenium nutritional status.

From a clinical perspective, serum SePP may serve as an accessible biomarker to help identify hemodialysis patients at higher risk of aortic stiffness and, by extension, adverse cardiovascular outcomes, supporting closer cardiovascular risk stratification in this population. Should future interventional studies confirm a causal role, selenium status assessment and correction could represent a modifiable target in the management of vascular risk in patients on MHD.

Future research should prioritize large, multicenter, longitudinal cohorts and randomized controlled trials to clarify the causal relationship between SePP and cardiovascular outcomes and to evaluate the efficacy and safety of selenium supplementation in patients on dialysis, utilizing aortic stiffness as a clinical endpoint. Studies are warranted to further elucidate the mechanisms underlying the impact of SePP on vascular pathology, including oxidative stress, inflammation, and endothelial dysfunction. Defining the optimal SePP range in patients on MHD and clarifying the significance of elevated SePP levels will provide important information to guide further clinical interventions.

## 5. Conclusions

In the present study of patients on MHD, serum SePP levels were inversely associated with aortic stiffness, assessed by cfPWV; the observed association remained independent after adjustment for clinical and biochemical factors and was further supported by penalized regression analyses. These findings support a potential association between SePP deficiency and arterial stiffening in patients on MHD.

## Figures and Tables

**Table 2:** Pearson correlation and multivariable linear regression analyses of clinical factors associated with log-transformed cfPWV.

Clinical Variables	Log-cfPWV (m/s)							
	Pearson Correlation		Multivariable Regression					
			Stepwise Model			Forced-Entry Model		
	*r*	*p* Value	B	β	*p* Value	B	β	*p* Value
Age (years)	0.203	0.017 *	0.002	0.163	0.032 *	0.002	0.179	0.022 *
Log-HD duration (months)	−0.110	0.198	—	—	—	—	—	—
Height (cm)	0.096	0.261	—	—	—	—	—	—
Pre-HD body weight (kg)	0.122	0.157	—	—	—	—	—	—
Post-HD body weight (kg)	0.113	0.187	—	—	—	—	—	—
Body mass index (kg/m^2^)	0.092	0.283	—	—	—	—	—	—
SBP (mmHg)	0.180	0.034 *	—	—	—	0.001	0.138	0.070
DBP (mmHg)	0.018	0.830	—	—	—	—	—	—
Hemoglobin (g/dL)	0.151	0.078	—	—	—	—	—	—
Log-Albumin (g/dL)	−0.022	0.797	—	—	—	—	—	—
Total cholesterol (mg/dL)	−0.104	0.226	—	—	—	—	—	—
Log-Triglyceride (mg/dL)	0.047	0.583	—	—	—	—	—	—
Log-Glucose (mg/dL)	0.189	0.027 *	—	—	—	0.075	0.065	0.392
BUN (mg/dL)	0.035	0.681	—	—	—	—	—	—
Creatinine (mg/dL)	−0.033	0.701	—	—	—	—	—	—
Total calcium (mg/dL)	0.084	0.330	—	—	—	—	—	—
Phosphorus (mg/dL)	0.011	0.884	—	—	—	—	—	—
Log-CRP (mg/dL)	0.246	0.004 *	0.049	0.183	0.017 *	0.044	0.162	0.034 *
Log-iPTH (pg/mL)	−0.144	0.093	—	—	—	—	—	—
Log-SePP (mg/L)	−0.437	<0.001 *	−0.283	−0.420	<0.001 *	−0.276	−0.409	<0.001 *
Urea reduction rate	−0.092	0.283	—	—	—	—	—	—
Kt/V (Gotch)	−0.099	0.250	—	—	—	—	—	—

Variables with skewed distributions were logarithmically transformed before analysis, including cfPWV, HD duration, albumin, triglycerides, glucose, CRP, iPTH, and SePP. Pearson correlation analysis was used to examine univariable associations between clinical variables and log-cfPWV. Variables significantly associated with log-cfPWV in Pearson correlation analysis were entered into the multivariable stepwise or forced-entry linear regression model (adjusted factors: age, SBP, log-glucose, log-CRP, and log-SePP). Variables not retained in the stepwise model or not included in the forced-entry model are indicated by em dashes. Values are presented as Pearson correlation coefficients (r), unstandardized regression coefficients (B), standardized regression coefficients (β), and *p* values. HD, hemodialysis; cfPWV, carotid-femoral pulse wave velocity; SBP, systolic blood pressure; DBP, diastolic blood pressure; BUN, blood urea nitrogen; CRP, C-reactive protein; iPTH, intact parathyroid hormone; SePP, selenoprotein P; Kt/V, fractional urea clearance index for urea. * *p* < 0.05 was considered statistically significant. Stepwise model: R^2^ = 0.262, adjusted R^2^ = 0.245, model *p* < 0.001. Forced-entry model: R^2^ = 0.286, adjusted R^2^ = 0.259, model *p* < 0.001.

**Table 3:** Multivariable and penalized logistic regression analyses of factors associated with aortic stiffness.

Factors	Multivariable		LASSO		Ridge		Elastic Net	
	OR (95% CI)	*p* Value	OR (95% CI)	*p* Value	OR (95% CI)	*p* Value	OR (95% CI)	*p* Value
SePP (mg/L)	0.676 (0.535, 0.853)	<0.001 *	0.708 (0.550, 0.870)	0.005 *	0.731 (0.621, 0.863)	0.005 *	0.719 (0.588, 0.871)	0.005 *
CRP (0.1 mg/dL)	1.077 (1.001, 1.159)	0.047 *	1.061 (1.000, 1.137)	0.055	1.056 (1.010, 1.111)	0.005 *	1.058 (1.003, 1.125)	0.035 *
DM	0.913 (0.286, 2.915)	0.878	1.000 (0.523, 2.286)	1.000	1.054 (0.533, 2.181)	0.860	1.000 (0.547, 2.226)	1.000
Hypertension	0.908 (0.231, 3.565)	0.890	1.000 (0.455, 3.298)	1.000	1.226 (0.550, 2.837)	0.600	1.038 (0.532, 3.108)	0.885
Age (years)	1.038 (1.002, 1.074)	0.036 *	1.032 (1.004, 1.076)	0.025 *	1.032 (1.007, 1.066)	0.005 *	1.031 (1.005, 1.070)	0.020 *
SBP (mmHg)	1.036 (1.000, 1.072)	0.050 *	1.029 (1.003, 1.060)	0.035 *	1.024 (1.007, 1.045)	0.015 *	1.027 (1.005, 1.050)	0.020 *
Glucose (mg/dL)	1.011 (0.995, 1.027)	0.184	1.009 (1.000, 1.020)	0.185	1.008 (0.999, 1.017)	0.095	1.009 (1.000, 1.018)	0.150

Values are presented as odds ratios (ORs) with 95% confidence intervals (CIs) and *p* values. Aortic stiffness was entered as the binary dependent variable. The multivariable and penalized logistic regression models included diabetes mellitus, hypertension, age, SBP, glucose, CRP, and SePP as covariates. Continuous predictors are expressed per 1-unit increase, except for CRP, which is expressed per 0.1 mg/dL increase. Diabetes mellitus and hypertension were entered as binary variables. Penalized logistic regression models included LASSO, Ridge, and Elastic Net regression. Penalized models were fitted after standardization for regularization, and ORs are shown on the original variable scale. For penalized logistic regression models, 95% CIs and *p*-values were estimated using stratified bootstrap resampling with 400 iterations. CI, confidence interval; CRP, C-reactive protein; DM, diabetes mellitus; OR, odds ratio; SBP, systolic blood pressure; SePP, selenoprotein P. * *p* < 0.05 was considered statistically significant.

**Table 4:** Discrimination and calibration performance of SePP for aortic stiffness.

Outcome	Events/Total	AUC (95% CI), *p* Value	Optimal Cutoff (mg/L)	Sensitivity	Specificity	Brier Score	Hosmer-Lemeshow, *p* Value
Aortic stiffness	56/138	0.674 (0.580–0.759) *p* < 0.001 *	3.26	0.571	0.720	0.217	0.339

The AUC was calculated using SePP as a single predictor. Because lower SePP levels were associated with aortic stiffness, the optimal cutoff was selected using the Youden index, with lower SePP levels indicating a higher risk. The Brier score and Hosmer-Lemeshow goodness-of-fit test were calculated from the logistic model using SePP alone. AUC, area under the receiver operating characteristic curve; CI, confidence interval; SePP, selenoprotein P. * *p* < 0.05 was considered statistically significant.

**Table 5:** Spearman’s correlation analysis between SePP and clinical variables.

Variables	Spearman Coefficient of Correlation	*p* Value
Age (years)	−0.025	0.769
HD duration (months)	0.129	0.131
Height (cm)	−0.150	0.079
Pre-HD body weight (kg)	−0.096	0.262
Post-HD body weight (kg)	−0.109	0.201
Body mass index (kg/m^2^)	−0.029	0.734
Carotid-femoral PWV (m/s)	−0.423	<0.001 *
Systolic blood pressure (mmHg)	−0.016	0.853
Diastolic blood pressure (mmHg)	−0.088	0.304
Hemoglobin (g/dL)	−0.241	0.004 *
Albumin (g/dL)	−0.025	0.770
Total cholesterol (mg/dL)	0.018	0.837
Triglyceride (mg/dL)	−0.134	0.118
Glucose (mg/dL)	−0.135	0.113
Blood urea nitrogen (mg/dL)	−0.063	0.460
Creatinine (mg/dL)	−0.018	0.831
Total calcium (mg/dL)	0.067	0.433
Phosphorus (mg/dL)	0.005	0.951
C-reactive protein (mg/dL)	−0.181	0.033 *
iPTH (pg/mL)	0.145	0.091
Urea reduction rate	−0.001	0.991
Kt/V (Gotch)	−0.004	0.967

Values are presented as Spearman’s rank correlation coefficients (ρ) and *p* values. SePP levels were analyzed using raw selenoprotein P concentrations. HD, hemodialysis; iPTH, intact parathyroid hormone; PWV, pulse wave velocity; SePP, selenoprotein P. * *p* < 0.05 was considered statistically significant (two-tailed).

## Data Availability

The data presented in this study are available on request from the corresponding author due to privacy and ethical restrictions related to patient confidentiality.

## Abbreviations

The following abbreviations are used in this manuscript:

AUC	Area under the receiver operating characteristic curve
CKD	Chronic kidney disease
cfPWV	Carotid-femoral pulse wave velocity
CRP	C-reactive protein
CV	Cardiovascular
CVD	Cardiovascular disease
iPTH	Intact parathyroid hormone
Kt/V	Fractional urea clearance index
LASSO	Least Absolute Shrinkage and Selection Operator
MHD	Maintenance hemodialysis
NAFLD	Non-alcoholic fatty liver disease
ROC	Receiver operating characteristic
OR	Odds ratio
SePP	Selenoprotein P
SBP	Systolic blood pressure
